# Large-scale multiple testing in genome-wide association studies via region-specific hidden Markov models

**DOI:** 10.1186/1471-2105-14-282

**Published:** 2013-09-25

**Authors:** Jian Xiao, Wensheng Zhu, Jianhua Guo

**Affiliations:** 1 Key Laboratory for Applied Statistics of MOE, School of Mathematics and Statistics, Northeast Normal University, Changchun 130024, China

## Abstract

**Background:**

Identifying genetic variants associated with complex human diseases is a great challenge in genome-wide association studies (GWAS). Single nucleotide polymorphisms (SNPs) arising from genetic background are often dependent. The existing methods, i.e., local index of significance (LIS) and pooled local index of significance (PLIS), were both proposed for modeling SNP dependence and assumed that the whole chromosome follows a hidden Markov model (HMM). However, the fact that SNP data are often collected from separate heterogeneous regions of a single chromosome encourages different chromosomal regions to follow different HMMs. In this research, we developed a data-driven penalized criterion combined with a dynamic programming algorithm to find change points that divide the whole chromosome into more homogeneous regions. Furthermore, we extended PLIS to analyze the dependent tests obtained from multiple chromosomes with different regions for GWAS.

**Results:**

The simulation results show that our new criterion can improve the performance of the model selection procedure and that our region-specific PLIS (RSPLIS) method is better than PLIS at detecting disease-associated SNPs when there are multiple change points along a chromosome. Our method has been used to analyze the Daly study, and compared with PLIS, RSPLIS yielded results that more accurately detected disease-associated SNPs.

**Conclusions:**

The genomic rankings based on our method differ from the rankings based on PLIS. Specifically, for the detection of genetic variants with weak effect sizes, the RSPLIS method was able to rank them more efficiently and with greater power.

## Background

At the present time, genome-wide association studies (GWAS) have become a very popular tool for identifying novel genetic variants for complex traits. GWAS typically tests hundreds of thousands of markers simultaneously, making it necessary to improve the power of large-scale multiple testing. Fortunately, the false discovery rate (FDR) for controlling such procedures, which was introduced in a seminal paper [1], is one of the most important methodological developments in multiple hypothesis testing and has played successful role in many large-scale multiple testing studies. Such studies include multi-stage clinical trials, microarray experiments, brain imaging studies, and astronomical surveys, amongst others [2-10]. Recently, the FDR approach has also been applied to GWAS [11]. Naturally, despite the increasing popularity of FDR, most of the traditional analytical methods for GWAS with FDR control have largely been proposed for individual single nucleotide polymorphism (SNP) analysis. However, because SNPs on the same chromosome are in local linkage disequilibrium (LD), which results in the complex dependence and correlation among large-scale tests, the traditional FDR controlling procedure for independent tests can potentially be conservative and lead to a loss of power. Therefore, it is important to consider FDR control for multiple testing procedures when the tests are dependent in GWAS.

Fortunately, Wei and Li pointed out that genomic dependency information could significantly improve the efficiency of analysis of large-scale genomic data [12,13]. We also expect that information about SNP dependency can be exploited to construct tests that are more efficient. From a biological point of view, SNP dependency is informative when constructing more efficient association tests, because when a SNP is associated with a disease, it is likely that the neighboring SNPs are also disease-associated (owing to the co-segregation). Therefore, when deciding the significance level of a SNP, its neighboring SNPs should be taken into account. Sun and Cai [14] proposed a local index of significance (LIS) controlling procedure that uses a hidden Markov model (HMM) to represent the dependence structure, and has shown its optimality under certain conditions and its strong empirical performance. Furthermore, this LIS procedure was extended to pooled local index of significance (PLIS) procedure for multiple-chromosome analysis [15], where the authors developed chromosome-specific HMMs for analysis of the SNP data arising from large-scale GWAS. Instead of HMM, Li [16] introduced a hidden Markov random field model (HMRFM) to account for LD when analyzing the SNP data from GWAS.

Therefore, the above methods are all based on the strong assumption that each chromosome follows a HMM or HMRFM. However, there are usually various LD patterns or haplotype blocks in the data, which result in heterogenity of dependencies among SNPs and variations in the disease risk rates of casual alleles in the different blocks. Hence, we suggest that the different blocks of each chromosome should follow different HMMs. Wei et al. [15] have stated that the development of a multiple testing procedure essentially involves two steps: ranking the hypotheses and choosing a cutoff along the rankings, where the ranking step is more fundamental. Obviously, modeling different regions by different HMMs can improve the efficiency of ranking. To this end, we should first identify change points for each chromosome, which can be used to divide the whole chromosome into smaller homogeneous regions. Specifically, we need to determine the number of change points as well as their locations on chromosomes.

In addition, the existing methods [14,15] assume that the observation variables follow a normal mixture distribution conditional on the latent variables in HMM, where the number of components is unknown in the normal mixture distribution. Sun and Cai [14] showed that the number of components should be determined by the likelihood-based Bayesian information criterion (BIC). However, BIC, as well as many other existing criterions, rely on a strong assumption that the observations are independent and require large sample sizes to reach their asymptotic consistency behavior. While in HMM, the observations are dependent such that the effective sample size for these criterions may be small.

In this paper, we first focus on the problem of how to infer simultaneously the number of components in a normal mixture distribution as well as the change points of each chromosome. We put forward a data-driven penalized criterion for model selection in HMM, and propose a sliding window-based improved version of the dimension jump method [17] to estimate this criterion. We then applied the dynamic programming (DP) algorithm to find multiple change points. The numerical results show that the proposed adaptive criterion has better performance than the original version. Second, we extended the approach of Wei et al. [15] to develop a testing procedure, which we have called region-specific PLIS (RSPLIS), for the analysis of different chromosomes with multiple regions. The numerical results show that RSPLIS outperforms PLIS in a disease association study. Our proposed procedure has been used to analyze the data from Daly et al. [18] for identifying Crohn’s disease-associated SNPs.

## Methods

First, Sun and Cai [14] developed a compound decision-theoretic framework for testing HMM-dependent hypotheses and presented an optimal testing procedure that can be used to analyze a single chromosome for SNP data. Second, Wei et al. [15] proposed a PLIS approach for multiple-chromosome analysis. They showed that under some regularity conditions, the PLIS procedure is valid and asymptotically optimal in the sense that it can control the global chromosome-wise FDR at the nominal level *α* and has the smallest false non-discovery rate (FNR) among all valid FDR procedures by combining the testing results from different chromosomes. In this section, we extend the chromosome-specific PLIS to the analysis of different chromosomes with multiple regions. In what following, we formulate our statistical model and elaborate the theoretical foundations of our method.

### Region-specific multi-HMM with change points and where the number of components known

In this subsection, we assume that we have known change points as well as the number of components in the normal mixture distribution. Let *w*_*c*_ and *m*_*c*_ denote the change point set and the number of components for chromosome *c*. Suppose the case-control genotype data are available from the *L*_*cr*_ SNPs in the *r*-th region of chromosome *c*, *c*=1,…,*C*, *r*=1,…,*R*_*c*_. We let *θrl*(*c*)=1 indicate that SNP *l* from region *r* of chromosome *c* is disease-associated and *θrl*(*c*)=0 otherwise. For each SNP, we first obtain a *p*-value by conducting a *χ*^2^-test to assess the association between the allele frequencies and the disease status, then we convert the *p*-value into a *z*-value *Zrl*(*c*) using the transformations proposed in Wei et al. [15] for further analysis. For chromosome *c*, let *θ*^(*c*)^={*θrl*(*c*);*l*=1,…,*L*_*cr*_,*r*=1,…,*R*_*c*_} and *Z*^(*c*)^={*Zrl*(*c*);*l*=1,…,*L*_*cr*_,*r*=1,…,*R*_*c*_}. In the following, we treat *θ*^(*c*)^ as the hidden variables and *Z*^(*c*)^ as the observed variables to consider HMMs using some assumptions.

First, we assume that the observed data are conditionally independent given the hidden states for the same region, and that different regions of the same chromosome are independent. Then we have

$$P(Z^{\left(c\right)}|\theta^{\left(c\right)},w_{c},m_{c})=\overset{R_{c}}{\underset{r=1}{\prod }}\overset{L_{\text{cr}}}{\underset{l=1}{\prod }}P(Z_{\text{rl}}^{\left(c\right)}|\theta_{\text{rl}}^{\left(c\right)},w_{c},m_{c}).\;\;\;$$

Furthermore, let $Z_{\text{rl}}^{\left(c\right)}|\theta_{\text{rl}}^{\left(c\right)},w_{c},m_{c}∼(1-\theta_{\text{rl}}^{\left(c\right)})F_{r0}^{\left(c\right)}+\theta_{\text{rl}}^{\left(c\right)}F_{r1}^{\left(c\right)}$, where $F_{r}^{\left(c\right)}=\{F_{r0}^{\left(c\right)},F_{r1}^{\left(c\right)}\}$ denotes the observation distribution for each SNP in region *r* of the chromosome *c*. For a non-associated SNP, we assume that the *z*-value distribution is a standard normal $F_{r0}^{\left(c\right)}=N(0,1)$ for all regions of chromosome *c*, and for a disease-associated SNP, the *z*-value distribution is a normal mixture, whereby $F_{r1}^{\left(c\right)}=\overset{m_{c}}{\underset{i=1}{\sum }}\xi_{i}N(\mu_{\text{ri}}^{\left(c\right)},{\sigma_{\text{ri}}^{\left(c\right)}}^{2})$, $\underset{i}{\sum }\xi_{i}=1$, and we assume that the number of components in the normal mixture is identical for all regions of chromosome *c*. The normal mixture model can approximate a large collection of distributions and has been widely used elsewhere [19-21].

Second, we assume that the hidden states $\theta_{r}^{\left(c\right)}$ and $\theta_{t}^{\left(c\right)}$ are independent for the different regions, *r* and *t*. For the *r*-th region of chromosome *c*, we assume that $\theta_{r}^{\left(c\right)}=\{\theta_{r1}^{\left(c\right)},\ldots ,\theta_{{\text{rL}}_{\text{cr}}}^{\left(c\right)}\}$ is distributed as a stationary Markov chain with a transition probability $a_{\text{rij}}^{\left(c\right)}=P(\theta_{r(l+1)}^{\left(c\right)}=j|\theta_{\text{rl}}^{\left(c\right)}=i)$. Let us denote *Λ*_*cr*_=(*arij*(*c*)) the transition matrix and $\pi_{\text{cr}}={\left(\pi_{r0}^{\left(c\right)},\pi_{r1}^{\left(c\right)}\right)}^{\prime }$ the stationary distribution, where $\pi_{r0}^{\left(c\right)}=a_{r10}^{\left(c\right)}/(a_{r10}^{\left(c\right)}+a_{r01}^{\left(c\right)})$ and $\pi_{r1}^{\left(c\right)}=1-\pi_{r0}^{\left(c\right)}$.

Let *ϕ*_*cr*_={(*μri*(*c*),*σri*(*c*),*ξ*_*i*_);*i*=1,…,*m*_*c*_}, then we denote *Ψ*_*cr*_=(*Λ*_*cr*_,*π*_*cr*_,*ϕ*_*cr*_) the collection of HMM parameters for *r*-th region of chromosome *c*. When *w*_*c*_ and *m*_*c*_ are known, the maximum likelihood estimate of the HMM parameters can be obtained using the expectation-maximization (EM) algorithm [14,22].

### Adaptive criterion-based partitioning (ACP) method for finding change points and the number of components

However, in practice, change points and the number of components in the normal mixture distribution are often unknown. In this subsection, for each chromosome, we will give an ACP method to conduct a model selection procedure for simultaneously finding *w*_*c*_ and *m*_*c*_.

#### Candidate change point set

To effectively reduce the huge space of competing change points and save computation time, our ACP method needs a candidate change point set in advance. Here, we use a haplotype block partition method [23] to obtain the haplotype-block boundary points for each chromosome, which can be collected as the candidate change point set. Because the minimum length value of block *L*_*min*_ should be pre-specified in their haplotype block partition method, here, we let *L*_*min*_ be 300 for all our analysis.

#### Adaptive criterion-based partitioning procedure

Simultaneously inferring *m*_*c*_ and *w*_*c*_ can be regarded as a model selection problem. To select a desired model, the commonly used methods are established base on the criterion of minimizing the penalized negative maximum likelihood (e.g. BIC). However, many other existing criterions including BIC, assume that the observations are independent, which is not true in HMM. As a result, the effective sample sizes may be small owing to strong dependence among the observations, and the existing criterions may suffer from a failure of consistency. A data-driven penalized criterion was proposed in the Gaussian and least-squares regression model selection for independent observations [17,24,25]. Especially, [25] used this adaptive criterion for variable selection and clustering in Gaussian mixtures model and showed that this adaptive criterion outperforms other criterions (e.g. BIC) for small sample sizes. Following their work, we propose a data-driven penalized criterion for dependent observations in HMM.

Let $w_{c}\subset w_{c}^{0}$ denote a change point set for chromosome *c*, |*w*_*c*_| be the number of the change points in *w*_*c*_, and *Ψ*_*c*_={*Ψ*_*cr*_;*r*=1,…,*R*_*c*_}, where $w_{c}^{0}$ is the candidate change point set for chromosome *c*. Then, we consider a penalized maximum likelihood criterion with the following form

(1) $$\begin{array}{l} \;{\text{crit}}_{\lambda_{c}}(m_{c},w_{c})=-\text{ln}\left(P(Z^{\left(c\right)}|{\hat{\Psi }}_{c},m_{c},w_{c})\right)+{\text{pen}}_{L_{c}}(w_{c},m_{c})\\ \;=-\text{ln}\left(\underset{\theta^{\left(c\right)}}{\sum }P(Z^{\left(c\right)},\theta^{\left(c\right)}|{\hat{\Psi }}_{c},m_{c},w_{c})P(\theta^{\left(c\right)}|{\hat{\Psi }}_{c},m_{c},w_{c})\right)\\ \;+\lambda_{c}D_{\left(m_{c},w_{c}\right)}, \end{array}$$

where ${\hat{\Psi }}_{c}$ is the maximum likelihood estimator of the parameters *Ψ*_*c*_ in HMMs for chromosome *c* using an EM algorithm, and the penalty function ${\text{pen}}_{L_{c}}(w_{c},m_{c})=\lambda_{c}D_{\left(m_{c},w_{c}\right)}$ is designed to avoid overfit problems. In this penalty function, *λ*_*c*_>0 is a tuning parameter to be chosen depending on sample size $L_{c}=\overset{R_{c}}{\underset{r=1}{\sum }}L_{\text{cr}}$ and $D_{\left(m_{c},w_{c}\right)}$ is the number of parameters in the model. Furthermore, in this paper, we have $D_{\left(m_{c},w_{c}\right)}=(|w_{c}|+1)D_{m_{c}}$, where $D_{m_{c}}$ is the number of parameters that only depend on *m*_*c*_. If we let $\lambda_{c}=\frac{\ln(L_{c})}{2}$, this penalty function becomes the penalty function of BIC for HMM.

Given a value of *λ*_*c*_ in the penalized criterion ${\text{pen}}_{L_{c}}(w_{c},m_{c})=\lambda_{c}D_{\left(m_{c},w_{c}\right)}$, we can find ${ŵ}_{c}$ and ${\hat{m}}_{c}$ to minimize ${\text{crit}}_{\lambda_{c}}(m_{c},w_{c})$ of equation 1 by running Algorithm 1.

Algorithm 1

In step 1, we need to give pre-specified values of *K*_*max*_ and *m*_*max*_ in advance, where *K*_*max*_ denotes the maximum value of the number of change points for each chromosome, and *m*_*max*_ is the maximum value of the number of component. As we know, the number of true change points is usually far less than the number of the candidate change points in practical applications, so we can give a smaller value for *K*_*max*_ to save computation time. For *m*_*max*_, Wei et al. [15] suggested that values of between four and six are usually chosen.

In step 2, following the methods of [26] and [27], we provide an optimal partitioning search method for change points to estimate ${ŵ}_{c,i}$ given *λ*_*c*_ and *m*_*c*_, which is, in essence, a dynamic program (DP) algorithm. The detailed procedure about the optimal partitioning search method is shown in Additional file 1.

However, in practice, *λ*_*c*_ is unknown and needs to be calibrated and estimated from the data themselves. Slope heuristics [24] as well as its generalization, dimension jump method [17], are practical and effective calibration algorithms to estimate the optimal penalty ${\text{pen}}_{L_{c},\text{opt}}(w_{c},m_{c})=\lambda_{c,\text{opt}}D_{\left(m_{c},w_{c}\right)}$. Here, we propose a sliding window-based dimension jump method to estimate *λ*_*c*,*opt*_, where the sliding window is used to avoid losing cases involving several successive jumps. When the width of the sliding window is 1, our proposed method becomes the dimension jump method of Wei et al. [17]. The following algorithm describes the detailed procedure for estimating *λ*_*c*,*opt*_.

Algorithm 2

At the end of Algorithm 2, we can obtain the estimation ${\hat{\lambda }}_{c,\text{opt}}$ of the *λ*_*c*,*opt*_. Having ${\hat{\lambda }}_{c,\text{opt}}$, we can then run Algorithm 1 to obtain ${\hat{m}}_{c,\text{opt}}$ and ${ŵ}_{c,\text{opt}}$ as well as the desired optimal model by minimizing ${\text{crit}}_{\lambda_{c}}(m_{c},w_{c})$ of Equation (1). At the same time, we can get ${\hat{\Psi }}_{c}$, the estimates of model parameters *Ψ*_*c*_ based on the optimal model, where *c*=1,…,*C*.

### Pooled FDR control procedure for different chromosomes with multiple regions

After each chromosome is divided into different regions by change points, it is desirable that that the global region- wide FDR can also be controlled by combining the test results from multiple regions of different chromosomes. In the following, we extend the chromosome-specific PLIS to the RSPLIS and operate the new procedure in three steps: 

*Step 1.* For chromosome *c* (*c*=1,…,*C*), we search the change points to divide the whole chromosome into multiple regions using the ACP method. For each region *r*, we can get ${\hat{\Psi }}_{\text{cr}}$ by using the EM algorithm from which we can calculate the plug-in LIS statistic ${\text{LIS}}_{\text{rl}}^{\left(c\right)}=P_{{\hat{\Psi }}_{\text{cr}}}(\theta_{\text{rl}}^{\left(c\right)}=0|Z_{r}^{\left(c\right)})$ for all regions of each chromosome by using the forward-backward algorithm [28].

*Step 2.*Combine and rank the plug-in LIS statistics from different regions of multiple chromosomes. Denote by *LIS*_(1)_,…,LIS_(*L*)_ the ordered values and *H*_(1)_,…,*H*_(*L*)_ the corresponding hypotheses, where $L=\overset{C}{\underset{c=1}{\sum }}\overset{R}{\underset{r=1}{\sum }}L_{\text{cr}}.$

*Step 3.*Reject all *H*_(*i*)_, *i*=1,…,*l*, where $l=\text{max}\{i:(1/i)\overset{i}{\underset{j=1}{\sum }}{\text{LIS}}_{\left(j\right)}\leq \alpha \}.$

We define FNR as the expected proportion of falsely accepted hypotheses. Under a compound decision-theoretic framework, the following theorem can verify that our RSPLIS is valid and asymptotically optimal. We provide the detailed proof of the theorem in Additional file 1.

#### Theorem 1

Consider the multi-region HMMs defined in section 'Region-specific multi-HMM with change points and where the number of components known’. Let LIS_(1)_,…,LIS_(*L*)_ be the ranked LIS values from all the regions of all chromosomes. Then, the RSPLIS procedure controls the global FDR at level *α*. In addition, the global FNR level of RSPLIS is *β*^∗^+*o*(1), where *β*^∗^ is the smallest FNR level among all valid FDR procedures at level *α*.

## Results

### Simulation study

In this section, we design the detailed simulation studies to illustrate the performance of our ACP method in model selection; thereafter we conducted simulation studies to compare the performance of the proposed RSPLIS with that of PLIS in GWAS. All the simulations that follow were replicated 100 times.

#### Simulations of the ACP method performance for model selection

Simulations in this subsection were conducted to compare the performance of our ACP method with that of BIC-based partitioning (BICP) method for selecting change points and the number of components. For simplicity, we consider a single chromosome that has five stationary regions. We assume each region has the same length *L*_0_ and set *L*_0_ equal to 600, 900 and 1200. The detailed simulation parameter settings are given in Table 1. With different parameter settings, we expect that the first two change points can be identified easily, while the last two change points are harder to be identified.

**Table 1:** Parameter settings of simulated data

**Region**	**Transition matrix**	**Stationary distribution**	**z-value distribution**	**z-value distribution**
			**(Null)**	**(No-null)**
1	$\left(\begin{array}{ll} 0.98 & 0.02\\ 0.20 & 0.80 \end{array}\right)$	(0.91, 0.09)	*N*(0,1)	0.1*N*(1.0,1)+0.9*N*(3.0,1)
2	$\left(\begin{array}{ll} 0.98 & 0.02\\ 0.95 & 0.05 \end{array}\right)$	(0.98, 0.02)	*N*(0,1)	0.8*N*(1.5,1)+0.2*N*(4.5,1)
3	$\left(\begin{array}{ll} 0.98 & 0.02\\ 0.45 & 0.55 \end{array}\right)$	(0.96, 0.04)	*N*(0,1)	0.4*N*(1.5,1)+0.6*N*(3.5,1)
4	$\left(\begin{array}{ll} 0.98 & 0.02\\ 0.15 & 0.85 \end{array}\right)$	(0.88, 0.12)	*N*(0,1)	0.2*N*(1.0,1)+0.8*N*(3.0,1)
5	$\left(\begin{array}{ll} 0.98 & 0.02\\ 0.15 & 0.85 \end{array}\right)$	(0.88, 0.12)	*N*(0,1)	0.2*N*(1.0,1)+0.8*N*(3.0,1)

In this simulation, we give the candidate change point set

$$w^{0}=\{300i;i=1,2,\ldots ,\frac{5L_{0}-300}{300}\},$$

which ensures that the true change point set {*iL*_0_;*i*=1,…,4}⊂*w*^0^. To compare the performance of the two methods, we used sensitivity and specificity as measures. Sensitivity is defined as the average proportions of the true change points which are correctly identified as change points over the 100 times and the specificity is defined as the average proportions of the false change points which are not identified as the change points over the 100 times. We set *K*_*max*_=8 and *m*_*max*_=5 for our ACP method. At the same time, *h* takes the values of 2, and 20 for the window.

The simulation results are summarized in Tables 2 and 3. From these tables, we can see that BICP misses most of the true change points and the true number of components. Moreover, we can also see our ACP has higher specificity than BICP. The reason for the poorer behavior of BICP may be related to the lack of independent observations in this experiment, so there may be a smaller effective sample size for BIC. In addition, based on the simulation results, we can see that the performance of our ACP is very good for *h*=2 and *h*=10, but is very poor for *h*=20, so we suggest *h* should not be more than 10 in practice.

**Table 2:** **The results of comparing ACP method with BICP method for selecting the true number of components*m=2***

**Length of region**	**Measures**	**ACP**			**BICP**
**(*L*)**		***h=2***	***h=10***	***h=20***	
600	Sensitivity	0.86	0.83	0.21	0.18
	Specifity	0.97	0.96	0.80	0.80
900	Sensitivity	0.83	0.80	0.13	0.16
	Specifity	0.96	0.96	0.78	0.79
1200	Sensitivity	0.81	0.78	0.12	0.15
	Specifity	0.95	0.94	0.78	0.79

**Table 3:** **The results of comparing ACP method with BICP method for selecting the true change point set {*iL*;*i*=1,…,4}**

**Length of region**	**Measures**	**ACP**			**BICP**
**(*L*)**		***h=2***	***h=10***	***h=20***	
600	Sensitivity	0.91	0.90	0.27	0.22
	Specifity	0.90	0.85	0.74	0.73
900	Sensitivity	0.68	0.67	0.13	0.13
	Specifity	0.86	0.85	0.81	0.83
1200	Sensitivity	0.42	0.29	0.08	0.08
	Specifity	0.89	0.82	0.86	0.87

#### HMM-based simulations for comparing RSPLIS with PLIS in GWAS

For simplicity, in this simulation, suppose there are two chromosomes (*c*=2) in total, each of which consists of two stationary regions, and each region has 2000 SNPs (*L*_*cr*_=2000,*r*=1,2,*c*=1,2). For each chromosome, we set *K*_*max*_=4, *m*_*max*_=3, and *h*=2 for our ACP method and gave the candidate change point set $w_{c}^{0}=\{300i;i=1,2,\ldots ,\frac{L_{c}-300}{300}\}$, *c*=1,2. The purpose of this simulation is to compare RSPLIS with PLIS by finding disease-associated SNPs while controlling the FDR at a pre-specified level *α*=0.1 for the two chromosomes (combining chromosomes 1 and 2). We conducted simulation studies in the following two cases.

##### Case 1

In this case, we varied the dependence parameters in transition matrices of HMM and kept the other parameters fixed, and then we investigated the behavior of RSPLIS and compare it with PLIS procedure to identify casual SNPs at the different disease risk levels. We used the parameter settings in Table 4, where we varied the degree of dependence among SNPs in region 2 by changing the value of *υ*_1_ (*υ*_1_=0,0.15,0.30,0.45). Furthermore, we let $\mu_{21}^{\left(c\right)}=\mu_{11}^{\left(c\right)}+1.5$, $\mu_{11}^{\left(2\right)}=\mu_{11}^{\left(1\right)}+0.5$ and varied the disease risk parameter $\mu_{11}^{\left(1\right)}$ from 0.5 to 2.0 with an increment of 0.5.

**Table 4:** **Parameter settings of simulated data for Case1**

**Chromosome**	**Region**	**Transition matrix**	**z-value distribution**	**z-value distribution**
**(*c*)**	**(*r*)**		**(Null)**	**(Non-null)**
1	1	$\left(\begin{array}{ll} 0.98 & 0.02\\ 0.03 & 0.97 \end{array}\right)$	*N*(0,1)	$N(\mu_{11}^{\left(1\right)},1)$
	2	$\left(\begin{array}{ll} 0.98 & 0.02\\ 0.03+\upsilon_{1} & 0.97-\upsilon_{1} \end{array}\right)$	*N*(0,1)	$N(\mu_{21}^{\left(1\right)}=\mu_{11}^{\left(1\right)}+1.5,1)$
2	1	$\left(\begin{array}{ll} 0.98 & 0.02\\ 0.05 & 0.95 \end{array}\right)$	*N*(0,1)	$N(\mu_{11}^{\left(2\right)}=\mu_{11}^{\left(1\right)}+0.5,1)$
	2	$\left(\begin{array}{ll} 0.98 & 0.02\\ 0.05+\upsilon_{1} & 0.95-\upsilon_{1} \end{array}\right)$	*N*(0,1)	$N(\mu_{21}^{\left(2\right)}=\mu_{11}^{\left(2\right)}+2.0,1)$

##### Case 2

In contrast to Case 1, to assess the performance of RSPLIS at the different disease risk levels, we varied the parameters of the *z*-value distribution while fixing the other parameters. We used the parameter settings in Table 5, where we varied the parameters of the z-value distribution by changing the value of *υ*_2_ (*υ*_2_=0.5,1,1.5,2). Furthermore, we let $\mu_{21}^{\left(c\right)}=\mu_{11}^{\left(c\right)}+\upsilon_{2}$, $\mu_{11}^{\left(2\right)}=\mu_{11}^{\left(1\right)}+0.5$ and varied the disease risk parameter $\mu_{11}^{\left(1\right)}$ from 0.5 to 2.0 with an increment of 0.5.

**Table 5:** **Parameter settings of simulated data for Case2**

**Chromosome**	**Region**	**Transition matrix**	**z-value distribution**	**z-value distribution**
**(*c*)**	**(*r*)**		**(Null)**	**(Non-null)**
1	1	$\left(\begin{array}{ll} 0.98 & 0.02\\ 0.025 & 0.975 \end{array}\right)$	*N*(0,1)	$N(\mu_{11}^{\left(1\right)},1)$
	2	$\left(\begin{array}{cc} 0.98 & 0.02\\ 0.025 & 0.975 \end{array}\right)$	*N*(0,1)	$N(\mu_{21}^{\left(1\right)}=\mu_{11}^{\left(1\right)}+\upsilon_{2},1)$
2	1	$\left(\begin{array}{cc} 0.98 & 0.02\\ 0.025 & 0.975 \end{array}\right)$	*N*(0,1)	$N(\mu_{11}^{\left(2\right)}=\mu_{11}^{\left(1\right)}+0.5,1)$
	2	$\left(\begin{array}{cc} 0.98 & 0.02\\ 0.025 & 0.975 \end{array}\right)$	*N*(0,1)	$N(\mu_{21}^{\left(2\right)}=\mu_{11}^{\left(2\right)}+\upsilon_{2},1)$

**Figure 1:**
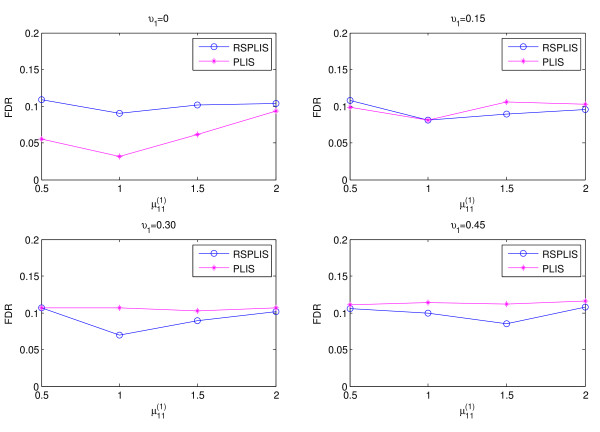
Shows that the FDR levels of the two methods are controlled at the level 0.10 asymptotically for four different parameter settings in Case 1.

**Figure 2:**
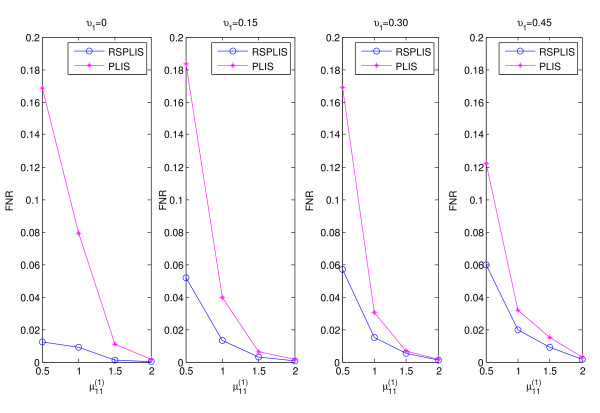
Shows the FNR of PLIS is much higher than that of RSPLIS for four different parameter settings in Case 1.

**Figure 3:**
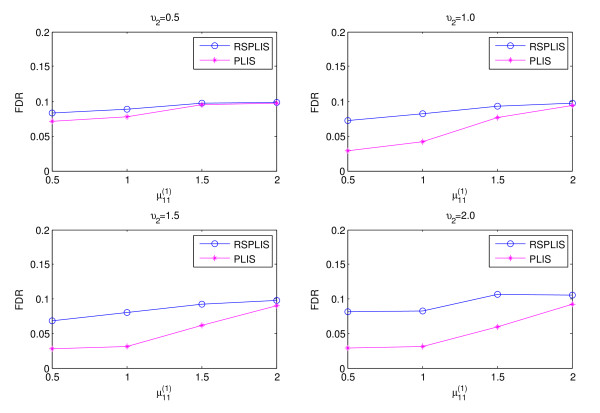
Shows the FDR levels of the two methods are controlled at 0.10 asymptotically for four different parameter settings in Case 2.

**Figure 4:**
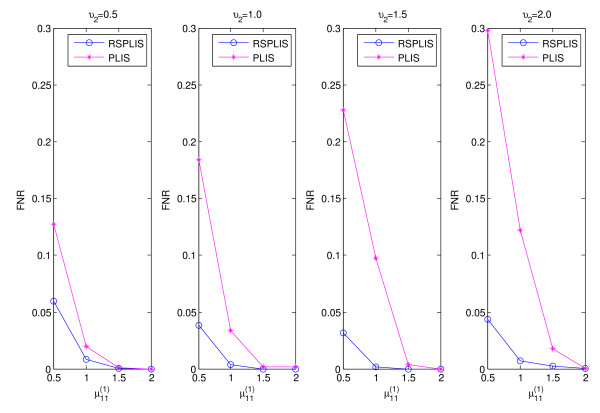
Shows the FNR of PLIS is much higher than that of RSPLIS for four different parameter settings in Case 2.

The simulation results are shown in Figures 1, 2, 3 and 4. From Figure 1 and Figure 3, we can obviously see that both RSPLIS and PLIS are well controlled at FDR level 0.1 asymptotically. Figure 2 and Figure 4 inform us that PLIS is dominated by RSPLIS for the power at Case 1 and Case 2, which indicates that our RSPLIS procedure is effective at dividing the chromosomes into smaller and more homogeneous regions by searching the change points. In addition, the difference in FNR levels (RSPLIS vs. PLIS) becomes smaller as $\mu_{11}^{\left(1\right)}$ increases for each model, which implies that RSPLIS is especially useful when the disease signals are weak.

**Figure 5:**
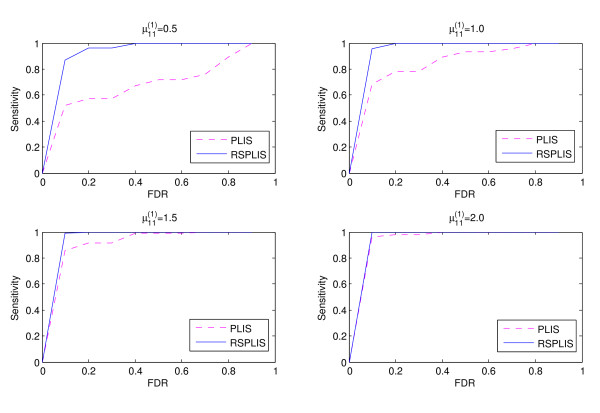
**Shows the ranking efficiency fo r*υ***_**1**_**=0 in Case 1: RSPLIS has higher sensitivity than PLIS at the same FDR level; there is a more dramatic improvement when the signals are weak (**$\mu_{11}^{\left(1\right)}$** is small).**

**Figure 6:**
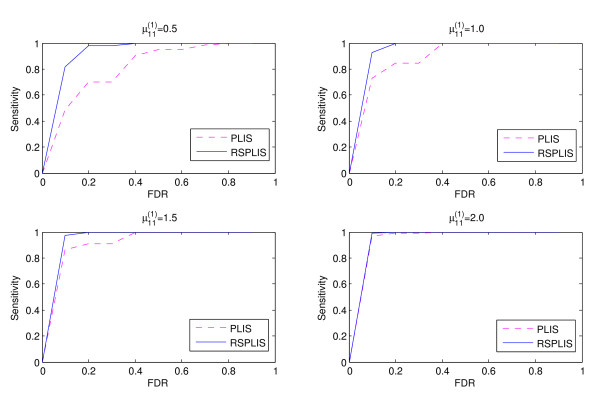
**Shows the ranking efficiency for *υ***_**1**_**=0 in Case2: RSPLIS has higher sensitivity than PLIS at the same FDR level; there is a more dramatic improvement when the signals are weak (**$\mu_{11}^{\left(1\right)}$** is small).**

To show that the higher power of RSPLIS is not gained to the detriment of a higher FDR level, we conducted a further simulation study. This study evaluated the sensitivities at different FDR levels for *υ*_1_=0,0.15,0.30,0.45 in Case 1, and *υ*_2_=0.5,1.0,1.5,2.0 in Case 2, where the sensitivities were calculated as the average proportions of correctly identified SNPs over the 100 replications. For the purpose of illustration, we have only listed the results for *υ*_1_=0.15 of Case 1 and *υ*_2_=1.0 of Case 2 in Figure 5 and Figure 6 respectively, because the other results were broadly similar. It is clear from Figures 5 and 6 that RSPLIS discovers more true disease-associated SNPs than PLIS at the same FDR level.

#### Genotype-based simulations for comparing RSPLIS with PLIS

This simulation evaluated the performance of selecting the *relevant* SNPs for RSPLIS and PLIS based on the genotype data set. In contrast to the simulation study in Subsection 'Application to the Daly data set’, we generated case-control genotype data with more realistic LD patterns. To this end, we constructed a genotype pool composed of genotypes from 60 samples for 23 chromosomes by randomly matching the pair of the known phased 120 haplotypes from the Illumina 550K. For simplicity, we only used SNPs selected from 2001-th SNP to 6000-th SNP of chromosome 7 and chromosome 15, respectively. Four SNPs were selected from each chromosome as the disease causal SNPs, each with a relative risk of 1.5. Specifically, the four SNPs, 400-th, 900-th, 1750-th, 3200-th, were chosen to be far away on chromosome 7, while the four other SNPs, 5600-th, 5604-th, 5608-th, 5612-th, were chosen based on their proximity to each other (i.e., separated by 3 SNPs) on chromosome 15.

For each subject, we first obtained the genotype, *X*, by drawing a genotype from the genotype pool at random. Using genotype *X*, we then simulated the disease status, *Y*, of this subject using the logistic regression model,

$$P\left(Y,=,1,|,X\right)=\frac{\text{exp}(\beta_{0}+\overset{8}{\underset{i=1}{\sum }}\beta_{i}X_{i})}{1+\text{exp}(\beta_{0}+\overset{8}{\underset{i=1}{\sum }}\beta_{i}X_{i})},$$

where *β*_0_=-9.5425 for a disease rate of 0.03, *β*_*i*_= log(1.5), for *i*=1,…,8. We repeated the sampling procedure until we obtained 1000 cases and 1000 controls are obtained. The eight disease casual SNPs were then removed from the simulated data set, and the 39 SNPs that contained the three adjacent SNPs on each side of the eight disease-causal SNPs were regarded as the relevant SNPs. We assessed the performance of the testing procedure by the selection rate of *relevant* SNPs, where the percentages of the true positives (sensitivity) selected by the top M SNPs could be calculated easily. We set *m*_*max*_=6, *h*=2, and *K*_*max*_=5 for the ACP method.

**Figure 7:**
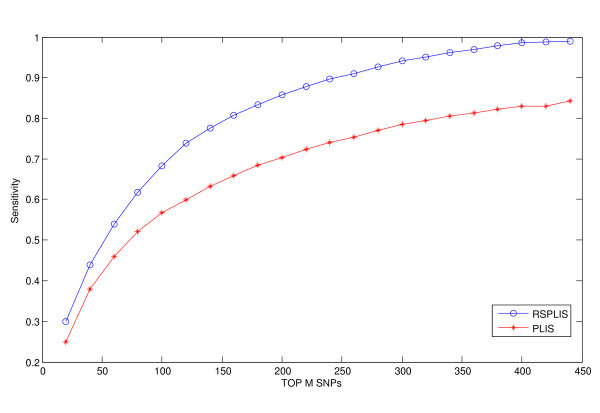
**The four SNPs in Chr 1 are far away from each other, while the four in Chr 2 are separated by only three SNPs.** We calculated the sensitivity selected by the top M SNPs, ranked by RSPLIS and by PLIS.

We have plotted the average sensitivity curves for comparisons of RSPLIS vs. PLIS in Figure 7. It is apparent that our RSPLIS dominates PLIS in ranking the *revelant* SNPs. In summary, these results show that exploiting the heterogeneous chromosomal regions and searching the change points to find chromosomal regions that are more homogeneous has improved the precision of RSPLIS in that the number of false positives has been reduced while the statistical power has increased.

### Application to the Daly data set

The data are derived from the 616 kilobase region of human chromosome 5q31 that may contain a genetic variant responsible for Crohn’s disease as determined by genotyping 103 common SNPs (>0.05 minor allele frequency) for 129 trios [18]. All offspring belong to the case population, while almost all parents belong to the control population. In the entire data, there are 144 cases and 243 controls. Daly et al. [18] have also shown that there are 11 blocks and strong LD between the SNPs and their neighboring SNPs in each block.

#### Model selection and estimation of HMM parameters

First, we used the *K*-Nearest neighbor method proposed by the R package [29] to impute the missing genotypes from the Daly et al. data [18]. Then, for each SNP, we obtained a *p*-value by conducting a *χ*^2^-test to assess associations between the allele frequencies and the disease status, furthermore, we get *z*-value by transforming *p*-value. We assumed that the null distribution is standard normal *N*(0,1) and the non-null distribution is a normal mixture $\overset{m_{c}}{\underset{i=1}{\sum }}\xi_{i}N(\mu_{\text{ri}}^{\left(c\right)},{\sigma_{\text{ri}}^{\left(c\right)}}^{2})$, where *c*=1 because there is only one chromosome in the Daly et al. data [18]. We used our ACP method to select the number of components and change points, where the parameters in HMMs were estimated by the EM algorithm. Thereafter, RSPLIS was used for multiple testing.

#### Data analysis

Because the Daly et al. data [18] have only 103 SNPs, we assumed only one change point for these data in our analysis. Thus, we took *L*_*min*_=20, *m*_*max*_=6, *h*=2 and took *K*_*max*_=1 for our ACP method. For the purpose of comparison, we also used the PLIS method to analyze the Daly et al. data [18]. The data [18] were collected to identify genetic variants conferring susceptibility to Crohn’s disease and nine SNPs were identified [30]. For the purpose of illustration, we only list here the LIS statistics and LIS ranks for the nine casual SNPs (Table 6). Based on the definition of LIS statistic given in Section 'Pooled FDR control procedure for different chromosomes with multiple regionsPooled FDR control procedure for different chromosomes with multiple regions multiple regions’, it is obvious that the smaller value of the LIS statistic means a larger probability that this SNP is associated with the disease. From Table 6, the rankings for eight causal SNPs illustrate that RSPLIS offers a marked improvement over PLIS, with the exception of locus 73. It is not surprising that not all of the nine causal SNPs are top ranked because non-casual SNPs that are strongly linked to the casual SNP may also be top-ranked. In summary, we can see that our method not only makes better rankings but also has smaller values for LIS statistics for most of the true casual SNPs. In addition, Table 6 shows that the LIS values obtained using RSPLIS are far lower than those obtained using PLIS. The reason may be that each region found by the ACP method has a smaller sample size for statistical inference in HMM, so this may affect the values obtained for the LIS statistic.

**Table 6:** Results of PLIS and RSPLIS for the known 9 casual SNPs in Daly data, which were reported as significant (Rioux et al., 2001)

**SNP name**	**SNP location**	**LIS statistics**		**LIS ranks**	
	**(th)**	**RSPLIS**	**PLIS**	**RSPLIS**	**PLIS**
IGR2055a-1	25	1.5994e-18	7.5411e-04	5	7
IGR2060a-1	26	1.7388e-18	8.5125e-04	7	9
IGR2063b-1	27	1.7389e-18	8.4605e-04	6	8
IGR2096a-1	33	8.0144e-18	4.1287e-03	39	51
IGR2198a-1	38	1.0731e-17	5.3388e-03	56	79
IGR2230a-1	48	3.4931e-18	2.1243e-03	11	13
IGR3081a-1	73	7.6344e-18	3.9276e-03	37	34
IGR3096a-1	77	3.7265e-18	2.3153e-03	12	14
IGR3236a-1	92	1.3554e-18	5.9574e-04	3	5

## Discussion

Large-scale multiple testing under dependence is holding promise in identifying genetic variants for GWAS. Previous research has focused on large-scale multiple testing under a HMM for a single chromosome ( [14,15]). In the present paper, we extended chromosome-specific PLIS to RSPLIS to analyze SNP data arising from large-scale GWAS by an adaptive penalized criterion. By dividing the whole chromosome into more homogeneous regions and conducting the extended pooling dependent testing procedure, we showed that the accuracy of a multiple testing procedure was improved when there are multiple change points along the whole chromosome. The numerical performances of our RSPLIS procedure were investigated using both simulated studies and real data analysis. The results showed that RSPLIS is more powerful than PLIS at identifying small effects in GWAS.

However, our method could be improved in several ways. In the present paper, we conducted large-scale multiple testing under a special form of dependence (HMM) for the hypotheses. Because complex LD structure(s) are usually stored in SNP data, the Markov chain may not be the most appropriate model for SNP dependence. Therefore, general forms of dependence such as the Markov random field should be considered in future, where the whole network is divided into a region-specific Markov random field network; this would improve the screening efficiency in GWAS.

Besides, the question of how to select an ideal candidate change point set is one issue that needs further consideration. Clearly, better prior knowledge can help us find the change point set to reduce the space of competing models in the model selection procedure. Thus, a better algorithm needs to be developed by using prior information to obtain the candidate change point set.

The computational complexity and feasibility of our RSPLIS approach for analyzing GWAS data that contain tens of thousands of SNPs merit further discussion. The RSPLIS method is made up of three independent procedures: a procedure for getting the candidate change point set, the adaptive criterion-based model selection procedure with HMM parameter estimations, and the pooled FDR control procedure for all the chromosomes with multiple regions, where the second procedure is the most time consuming. Fortunately, our method runs the second procedure chromosome-by-chromosome, which facilitates parallel computing. For each chromosome, say, the *c*-th chromosome, the computational complexity for three procedures is *O*(*Lmin*2*L*_*c*_), $O(8{\text{TK}}_{\text{max}}^{2}m_{\text{max}}|w_{c}^{0}|L_{c})$, and *O*(*L*_*c*_ log(*L*_*c*_)) respectively, where *L*_*c*_ denotes the number of SNPs on the *c*-th chromosome, $\left|w_{c}^{0}\right|$ denotes the number of the change points in candidate change point set $w_{c}^{0}$, *T*=50 in our Algorithm 2, and *m*_*max*_ is usually chosen between four and six [15]. In addition, our method is very flexible, which allows users to set the minimum length value of block (*L*_*min*_) as well as the maximum value of the number of change points (*K*_*max*_) in large-scale GWAS. Based on our simulation studies, with the setting, *L*_*min*_=300, *K*_*max*_=5, *T*=50, and *m*_*max*_=6, it took about 40 min for our RSPLIS procedure to analyze 8000 SNPs from two chromosomes. We expect that the running time for large-scale GWAS is still acceptable because we can use parallel computing for each chromosome.

## Conclusions

In this paper, we first modeled the observed dependent SNP data via region-specific multiple HMMs divided by change points, where we developed a novel data-driven penalized criterion combined with the DP algorithm to find change points. Second, we proposed a RSPLIS method to conduct the dependent tests from multiple chromosomes with different regions for GWAS. Finally, we have shown the numerical performances of the RSPLIS procedure using both simulated studies and analysis of a real data set.

## Availability

Matlab and R code for RSPLIS can be accessed at http://math.nenu.edu.cn/faculty/wszhu/softwares/RSPLIS.html. This site contains the program files and code introduction.

## Competing interests

The authors declare that they have no competing interests.

## Authors’ contributions

Designed the experiments: JX, WZ; Performed the experiments: JX; Wrote the paper: JX, WZ and JG. All authors contributed to the analysis, read, and approved the final manuscript.

## Supplementary Material

Additional file 1 **The derivation of the dynamic program algorithm and the proof of theorem1.** Additional file 1 contains the derivation of the dynamic program (DP) algorithm for the step 2 in Algorithm 1, the derivation of the RSPLIS procedure and proof of theorem 1. Click here for file
