# Supplementary material for: Large-scale multiple testing in genome-wide association studies via region-specific hidden Markov models
Source: BMC Bioinformatics. 2013 Sep 25;14:282. doi: 10.1186/1471-2105-14-282 (PMC3850654; doi:10.1186/1471-2105-14-282)
Supplement: Additional file 1 — The derivation of the dynamic program algorithm and the proof of theorem1. Additional file 1 contains the derivation of the dynamic program (DP) algorithm for the step 2 in Algorithm 1, the derivation of the RSPLIS procedure and proof of theorem 1. [file 1471-2105-14-282-S1.pdf]

Additional File 1

The derivation of the dynamic program algorithm for the step 2 in Algorithm 1

In the following, we will give a supplementary discussion about the step 2 in Algorithm 1 and the detailed derivations for the dynamic program algorithm. Firstly, we give some notes. Let $[q_i : q_j]$ denote a region from the locus q_i to the locus q_j and $\ln(P(Z^{(c)}_{[q_i:q_j]}|\hat{\Psi}_c, m_c, w_c))$ be the maximum log-likelihood about the data from $[q_i : q_j]$ of the whole observes $Z^{(c)}$, where $q_i, q_j \in\ w_c,\ \ q_0 = 0 < q_i < q_j < q_{(|w_c|+1)} = L_c,\ 0 < i < j < |w_c|+1$. Then we define the dimension $D_{(m_c,w_c)}$ as $(|w_c| + 1)D_{m_c}$, where D_{m_c} is the number of parameters only depending on m_c. In our model, $D_{m_c} = 3m_c + 2$ for chromosome c. Thus, given $m_c = i$ and w_c, the objection function of the step 2 in algorithm 1 becomes:

$$
\begin{aligned}
&-\ln(P(Z^{(c)}|\hat{\Psi}_c, m_c = i, w_c)) + \lambda_c D_{(m_c=i,w_c)} \\
&= \sum_{k=1}^{|w_c|+1} [-\ln(P(Z^{(c)}_{[(q_{k-1}+1):q_k]}|\hat{\Psi}_c, m_c = i, w_c))] + \lambda_c D_{(m_c=i)}].
\end{aligned}
$$

Then given $m_c = i$ and let $w_c[ii : jj]$ denote the change point set with the last region to be $[ii : jj]$, we define the score function for the change point set $w_c[ii : jj]$

$$
\begin{aligned}
\Delta^{w_c[ii:jj]}_{(m_c=i)} = &\sum_{k=1}^{|w_c[ii:jj]|} [-\ln(P(Z^{(c)}_{[(q_{k-1}+1):q_k]}|\hat{\Psi}_c, m_c = i, w_c)) + \lambda_c D_{(m_c=i)}] \\
&- \ln(P(Z^{(c)}_{[(ii+1):jj]}|\hat{\Psi}_c, m_c = i, w_c)) + \lambda_c D_{(m_c=i)}.
\end{aligned}
$$

Next, we define $\Delta^{K_c}_{(m_c=i)}[ii : jj]$ to be the minimum score of a change point set with the last region to be $[ii : jj]$ and the number of change points to be K_c

$$
\Delta^{K_c}_{(m_c=i)}[ii : jj] = \min_{w_c[ii:jj],|w_c[ii:jj]|=K_c} \{\Delta^{w_c[ii:jj]}_{(m_c=i)}\}.
$$

Thus, given $m_c = i$ and $K_c \leq K_{max}$, apply dynamic programming theory, we can get the following recursion

$$
\Delta^{K_c}_{(m_c=i)}[e + 1 : L_c] = \min_{w^1_{c,K_c} \leq f \leq e-1} \{\Delta^{K_c-1}_{(m_c=i)}[f + 1 : e] + \lambda_c D_{(m_c=i)}\} + \lambda_c D_{(m_c=i)},
$$

where $e, f \in w^1_c$, $w^1_c = \{0\} \cup w^0_c = \{w^1_{c,1}, w^1_{c,2}, \ldots, w^1_{c,|w^1_c|}\}$ with the bound that if $i < j$, $w^1_{c,i} < w^1_{c,j}$ holds.

Given λ_c and $m_c = i$, using the above recursion, we can design a dynamic programming algorithm to find the optimal change point set $\hat{w}_{c,t,i}$ in step 2 of Algorithm 1.

The derivation of the RSPLIS procedure and proof of theorem 1

The derivation of the RSPLIS procedure

The derivation involves three steps:

(i) making connections between the multiple testing and weighted classification problems;

(ii) derive an oracle procedure for FDR control;

(iii) develop a data-driven procedure that mimics the oracle procedure.

Let β be the relative cost of a false positive to a false negative. Consider a weighted classification problem with loss function

$$\mathcal{L}_\beta(\theta,\delta)=\frac{1}{L}\sum_c\sum_r\sum_l\{\beta(1-\theta_{rl}^{(c)})\delta_{rl}^{(c)}+\theta_{rl}^{(c)}(1-\delta_{rl}^{(c)})\}$$

where $L=\sum_{c=1}^{C}L_c$ is the total number of SNPs from all chromosomes. Under mild conditions, the multiple testing problem is equivalent to a weighted classification problem. Specifically, let $\mathcal{U}_\alpha$ be the collection of all α-level FDR procedures of the form $\delta=\mathrm{I}_{(\mathrm{T}<\mathcal{C}\mathbf{1})}$. Suppose that the classification risk with the above loss function is minimized by $\delta^\beta\{\mathrm{T},\mathcal{C}(\beta)\}$, so that T is optimal in the weighted classification problem. If $\mathrm{T}\epsilon\ \mathcal{T}$, then T is also optimal in the multiple testing problem, in the sense that for each FDR level α, there exists a unique $\beta(\alpha)$, and hence $\mathcal{C}\beta(\alpha)=\mathcal{C}(\alpha)$, such that $\delta^{\beta(a)}\{\mathrm{T},\mathcal{C}(\alpha)\}$ controls the FDR at level α with the smallest FNR level among all testing rules in $\mathcal{U}_\alpha$.

The optimal classification rule that minimizes $\mathrm{R}_\beta=E(\mathcal{L}_\beta(\theta,\delta))$ is $\delta(\beta,\frac{1}{\beta})=(\delta_{rl}^{(c)})$, where $\delta_{rl}^{(c)}=P_{\Psi_{cr}}(\theta_{rl}^{(c)}=0|Z_r^{(c)})/P_{\Psi_{cr}}(\theta_{rl}^{(c)}=1|Z_r^{(c)})$ and $\delta_{rl}^{(c)}=\mathrm{I}_{(\beta(Z_{rl}^{(c)})<1/\beta)}$. Note that $\beta_{rl}^{(c)}$ is strictly increasing in $\mathrm{LIS}_{rl}^{(c)}$, the optimal testing procedure is of the form

$$\delta(\mathrm{LIS},\mathcal{C}\mathbf{1})=\{\mathrm{I}_{(\mathrm{LIS}_{rl}^{(c)}<\mathcal{C})}:c=1,\dots,C;r=1,\dots,R_c;l=1,\dots,L_{cr}\}.$$

Now the question is how to determine the optimal cutoff $\mathcal{C}_{opt}$ for a given FDR level a. Note that for a given threshold $\mathcal{C}$, the FDR level of RSPLIS is

$$\begin{aligned}\mathrm{FDR}(\mathcal{C})&=E\{\frac{\sum_c\sum_r\sum_l(1-\theta_{rl}^{(c)})\delta_{rl}^{(c)}}{(\sum_c\sum_r\sum_l\delta_{rl}^{(c)})\vee 1}\}\\&=E[\frac{1}{(\sum_c\sum_r\sum_l\delta_{rl}^{(c)})\vee 1}\sum_c\sum_r\sum_l\mathrm{I}_{(\mathrm{LIS}_{rl}^{(c)}<t)}\mathrm{LIS}_{rl}^{(c)}].\end{aligned}$$

From the above expression we can see that the group labels c and r are no longer needed and hence are dropped. Suppose the total number of rejections from all groups is RN, then according to the law of large numbers,

$$\mathrm{FDR}=\frac{1}{\mathrm{RN}}\sum_{i=1}^{\mathrm{RN}}\mathrm{LIS}_{(i)}+o(1).$$

It is straightforward to see that we should choose the largest RN such that

$$\frac{1}{\mathrm{RN}}\sum_{i=1}^{\mathrm{RN}}\mathrm{LIS}_{(i)}\le\alpha.$$

Thus we have derived the RSPLIS procedure.

Proof of Theorem 1

(i) Validity. Let RN be the number of rejections by the RSPLIS procedure. Note that this is a pooled analysis, we neglect the group label c and r.

$$
\begin{aligned}
\mathrm{FDR}_{\mathrm{RSPLIS}} &= E\{\frac{\sum_{i=1,\ldots,L}\delta_i(1-\theta_i)}{(\sum_{i=1,\ldots,L}\delta_i)\vee 1}\} \\
&= E[\frac{1}{(\sum_{i=1,\ldots,L}\delta_i)\vee 1}\sum_{i=1,\ldots,L}E\{\delta_i(1-\theta_i)|Z\}] \\
&= E[\frac{1}{(\sum_{i=1,\ldots,L}\delta_i)\vee 1}\sum_{i=1,\ldots,L}\delta_i\mathrm{LIS}_i] \\
&= E\{\frac{1}{\mathrm{RN}\vee 1}\sum_{i=1,\ldots,\mathrm{RN}}\mathrm{LIS}_i\}.
\end{aligned}
$$

The result follows by noting that for all realizations of Z, Our RSPLIS procedure guarantees that

$$\frac{1}{\mathrm{RN}\vee 1}\sum_{i=1}^{\mathrm{RN}}\mathrm{LIS}_i \leq \alpha.$$

(ii) Asymptotic optimality. The asymptotic optimality can be shown without essential difficulty by generalizing the proof of Theorem 6 in Sun and Cai (2009) (for a single Markov chain). We refer to Sun and Cai (2009) for more technical details.
